# Multiprofessionelle Perspektiven auf die intersektorale Zusammenarbeit für die Prävention des postoperativen Delirs bei älteren Patient:innen – eine qualitative Studie

**DOI:** 10.1007/s00391-025-02442-4

**Published:** 2025-05-05

**Authors:** Simon Krutter, Chiara Muzzana, Maria Flamm, Bernhard Iglseder, Giuliano Piccoliori, Ingrid Ruffini, Patrick Kutschar, Dietmar Ausserhofer

**Affiliations:** 1 Institut für Pflegewissenschaft und -praxis, Paracelsus Medizinische Privatuniversität, Strubergasse 21, 5020 Salzburg, Österreich; 2 Claudiana Research, Landesfachhochschule für Gesundheitsberufe „Claudiana“, Bozen, Italien; 3 Universitätsklinik für Geriatrie, Universitätsklinikum Salzburg, Christian Doppler Klinik, Salzburg, Österreich; 4 Institut für Allgemein‑, Familien- und Präventivmedizin, Paracelsus Medizinische Privatuniversität, Salzburg, Österreich; 5 Abteilung für Geriatrie, Krankenhaus Meran, Südtiroler Sanitätsbetrieb, Meran, Italien; 6 Institut für Allgemeinmedizin und Public Health „Claudiana“, Bozen, Italien; 7 Zentrum für Public Health und Versorgungsforschung, Paracelsus Medizinische Privatuniversität, Salzburg, Österreich

**Keywords:** Postoperatives Delir, Interprofessionelle Zusammenarbeit, Prävention, Extra- und intramurale Versorgung, Qualitative Methoden, Postoperative delirium, Interprofessional collaboration, Prevention, Outpatient and inpatient settings, Qualitative methods

## Abstract

**Hintergrund:**

Für die Prävention des postoperativen Delirs (POD) bei älteren Patient:innen ist die interprofessionelle Zusammenarbeit aller am Versorgungskontinuum beteiligten Healthcare Professionals (HCP) zentral.

**Ziel:**

Die Studie geht der Frage nach, wie die HCP die interprofessionelle Prävention des POD bei älteren Patient:innen im Versorgungskontinuum erleben, und welche Herausforderungen und Änderungswünsche sie an die intersektorale Kommunikation und Zusammenarbeit zur Prävention des POD haben.

**Methoden:**

Diese multizentrische Studie wurde mit einem qualitativen Forschungsansatz durchgeführt. Zur Exploration der Erfahrungen der HCP wurden in Salzburg und der Region Südtirol insgesamt 21 semistrukturierte Experteninterviews mit Allgemeinmediziner:innen, Geriater:innen, Anästhesist:innen, Chirurg:innen und Pflegefachpersonen geführt und inhaltsanalytisch ausgewertet.

**Ergebnisse:**

Die Ergebnissen zeigen, dass die HCP Maßnahmen zur Prävention des POD setzen, diese sich aber primär auf die Identifikation von Risikopatient:innen im intramuralen Bereich beschränken. Informationen zu den Risikofaktoren werden interdisziplinär und intersektoral nicht systematisch ausgetauscht. Strukturen und Prozesse zu Kommunikation und Zusammenarbeit in der Prävention des POD über das Versorgungskontinuum hinweg sind kaum vorhanden.

**Schlussfolgerungen:**

Zur Prävention des POD bei älteren Patient:innen sollten vermehrt Anstrengungen unternommen werden, die intersektorale, interprofessionelle Zusammenarbeit und Kommunikation der beteiligten HPC zu verbessern. Ein gemeinsames Instrument zu systematischer Erfassung und Dokumentation der relevanten Risikofaktoren über das Versorgungskontinuum hinweg könnte dabei die Prävention des POD unterstützen.

**Zusatzmaterial online:**

Zusätzliche Informationen sind in der Online-Version dieses Artikels (10.1007/s00391-025-02442-4) enthalten.

## Abstract (en)

**Background:**

The prevention of postoperative delirium (POD) in older adults requires interprofessional collaboration among all healthcare professionals (HCP) involved in the continuum of care.

**Objective:**

This study investigated the question of how HCPs experience the interprofessional prevention of POD in older adults within the continuum of care. Additionally, it examined the challenges and desired changes in the intersectoral communication and collaboration for prevention of POD.

**Methods:**

This multicenter study was carried out with a qualitative research approach. To explore the experiences of HCPs a total of 21 semistructured expert interviews were conducted with general practitioners, geriatricians, anesthesiologists, surgeons and nurses across the 2 study regions Salzburg County and South Tyrol. The interviews were analyzed using content analysis.

**Results:**

The results show that the interviewed HCPs already implement measures to prevent POD but these predominantly focus on identifying patients at risk in the inpatient sector. Information about risk factors is not systematically shared between disciplines or across the intersectoral continuum of care. Structures and processes for intersectoral communication and collaboration in the prevention of POD over the continuum of care are largely missing.

**Conclusion:**

To improve the prevention of POD in older adults efforts should be made to enhance intersectoral and interprofessional collaboration and communication between the HCPs involved. The development of a shared instrument for the systematic identification and documentation of relevant risk factors across the continuum of care could support the prevention of POD.

## Hintergrund

Das postoperative Delir (POD) ist mit einer Prävalenz bis zu 50 % [2, 20] eine häufige Komplikation bei älteren Patient:innen mit orthopädischen und chirurgischen Eingriffen. Das POD ist mit erhöhter Mortalität, Verweildauer und Behandlungskosten assoziiert und zeigt sich klinisch wie ökonomisch als ein negatives Ereignis in der Versorgung hospitalisierter Patient:innen. In der Literatur werden prädisponierende (z. B. kognitive Beeinträchtigung) und auslösende Risikofaktoren (z. B. Operationsdauer) für das POD bei älteren Patient:innen beschrieben [6, 12]. Für die Prävention im perioperativen Versorgungskontinuum sind Screening und Assessment von Risikofaktoren besonders hervorzuheben [15].

Die interprofessionelle Kommunikation und Zusammenarbeit stellt eine essenzielle Voraussetzung für die erfolgreiche Umsetzung empfohlener Präventions- und Behandlungsmaßnahmen dar [1, 14]. Diesbezügliche Mängel sind häufig zentrale Barrieren für die Umsetzung eines umfassenden Präventions- und Behandlungsprogramm des POD [5]. Interprofessionelle Delir-Teams bestehen mindestens aus Ärzt:innen und Pflegefachpersonen, immer häufiger auch mit Beteiligung von Geriater:innen, Orthopäd:innen, Physio- sowie Ergotherapeut:innen [10].

Ein Großteil der Evidenz zu Prävention und Behandlung des POD bezieht sich auf die intramurale Versorgung [10]. Gleichzeitig sind Allgemeinmediziner:innen auch bei orthopädischen und chirurgischen Eingriffen häufig die ersten Ansprechpartner:innen für ältere Patient:innen. Bislang gibt es kaum Studien zum POD, welche den extramuralen Bereich miteinbeziehen [18]. Eine intersektorale Versorgung der geriatrischen Patient:innen ist notwendig, um den Informationsfluss zu potenziellen Risikofaktoren im perioperativen Versorgungskontinuum zu gewährleisten und interdisziplinäre und -professionelle Präventionsmaßnahmen frühzeitig umzusetzen [16].

Diese qualitative Studie ist Teil einer multizentrischen, multimethodischen Studie zur Entwicklung eines „Geriatrischen Delir-Passes (GeDePa)“, der die Risikofaktoren bei älteren Patient:innen mit elektiven Eingriffen extra- und intramural erfassen, die interprofessionelle Kommunikation und Zusammenarbeit fördern und die Patient:innen im Versorgungskontinuum begleiten soll. Die vorliegende Forschungsarbeit geht den folgenden Forschungsfragen nach:

1. Wie erleben die Healthcare Professionals (HCP) die intersektorale und interprofessionelle Prävention des POD bei älteren Patient:innen?
2. Welche Herausforderungen werden im Versorgungskontinuum wahrgenommen?
3. Welche Erwartungen an die intersektorale Kommunikation und Zusammenarbeit zur POD-Prävention bestehen?

## Methoden

Zur Erfassung der multiprofessionellen Erfahrungen und Sichtweisen der HCP wurden qualitative, semistrukturierte Experteninterviews angewendet.

### Sampling und Rekrutierung

Mittels kriterienbezogener Rekrutierung wurden an den Studienorten Salzburg und Südtirol gezielt klinische Expert:innen, die an der Versorgung geriatrischer Patient:innen mit elektiven Eingriffen im extra- und intramuralen Bereich beteiligt sind, aus den Professionen Allgemeinmedizin, Geriatrie, Anästhesie sowie Pflege eingeschlossen. Weitere Einschlusskriterien waren eine mindestens 3‑jährige praktische Berufserfahrung sowie eine schriftlich informierte Zustimmung zur Interviewteilnahme. Das Sampling erfolgte als Gelegenheitsstichprobe im Rahmen einer zweckmäßigen Auswahl mithilfe von Gatekeeper:innen aus der Praxis (z. B. Pflegedirektion, ärztliche Leitung geriatrischer Abteilungen), wobei auf eine vergleichbare Verteilung an den beiden Studienorten geachtet wurde. Bis auf 2 Ärzt:innen nahmen alle angefragten HCP an den Interviews teil. Im Verlauf wurden auch Schritte eines theoretischen Sampling gesetzt und Chirurg:innen eingeschlossen.

### Datenerhebung

Die leitfadengestützten Experteninterviews (s. Auszug aus Leitfaden im Zusatzmaterial online: ESM 1) wurden zwischen Dezember 2023 und März 2024 in deutscher oder italienischer Sprache von einem/r Projektmitarbeiter:in in Praxen, Besprechungsräumen an Kliniken oder Hochschulen durchgeführt und dauerten durchschnittlich 33 min (23–46 min). Die Interviews wurden tontechnisch aufgezeichnet, systematisch schriftsprachlich transkribiert und pseudonymisiert.

### Datenanalyse

Die Auswertung erfolgte mittels strukturierender qualitativer Inhaltsanalyse nach Mayring [7] mit dem Ziel, die Aussagen der Interviewten thematisch zu systematisieren und inhaltlich zusammenzufassen. Für die Auswertung wurden vorab deduktive Kategorien festgelegt, die sich an den Themen des Interviewleitfadens orientierten. Die im Laufe der Analyse aus den Daten gebildeten induktiven Kategorien spiegeln insbesondere die disziplinären und sektoralen Sichtweisen der Interviewten wieder. Zwei Forschende (CM, SK) werteten mittels MAXQDA 22 (VERBI Software) die Daten aus, wobei es im Rahmen des konsensuellen Codierens zu einer gemeinsamen Überprüfung der Codierungen mit zwei weiteren Forschenden (DA, PK) kam. Dabei wurde zugleich auch das Kategoriensystem präzisiert, und es erfolgte eine kritische Diskussion im Sinne der interpretativen Argumentationsabsicherung.

### Geltungsbegründung und ethische Reflexion

Die Erhebung und Auswertung der Daten orientierten sich an Gütekriterien qualitativer Forschung nach Mayring [7]. Die Studie hat ein positives Votum der Ethikkommission der SABES (Südtirol) (ref. 17-2023) erhalten und wurde von der Ethikkommission des Landes Salzburgs (ref.: 415-EALL/4/158/2-2023) als unbedenklich eingestuft.

## Ergebnisse

### Stichprobe

Insgesamt wurden 21 Interviews geführt (*n* = 13 Salzburg, *n* = 8 Südtirol) mit ärztlichen (*n* = 16) und pflegerischen HCP (*n* = 5), die mit einer durchschnittlichen Berufserfahrung von 18,7 Jahren als erfahren und mit durchschnittlich 7,8 Jahren in der derzeitigen Arbeitsstelle auch als gut informiert über die regionalen Strukturen und Prozesse der Versorgung zu bezeichnen sind. Entlang des perioperativen Versorgungskontinuums konnten dabei Akteure aller beteiligten Professionen eingeschlossen werden: Hausärzt:innen (*n* = 6), Geriater:innen (*n* = 4), Anästhesist:innen (*n* = 4), Chirurg:innen (*n* = 2) und Pflegende (*n* = 5; Tab. 1).

**Tab. 1:** Beschreibung des Sample

Stichprobengröße	*n* = 21
Geschlecht	M = 11 W = 10
Berufsbezeichnung (Profession)	Ärzt:innen = 16 Pflegefachpersonen = 5
Fachdisziplin	Hausärzt:in = 6 Geriater:in = 4 Anästhesist:in = 4 Chirurg:in = 2 Pflegefachperson = 3 Pflegeexpert:in APN = 2
Berufserfahrung (insgesamt)	⌀ 18,7 Jahre
Berufserfahrung (derzeitige Arbeitsstelle)	⌀ 7,8 Jahre

Die Ergebnisse zu den Erfahrungen, Herausforderungen und Erwartungen der HCP in der Prävention des POD bei geriatrischen Patient:innen mit elektiven Eingriffen umfassen 5 zentrale Themenbereiche (Tab. 2).

**Tab. 2:** Zentrale Themen der Analyse

1.	Maßnahmen der Früherkennung von Risikopatient:innen im Versorgungskontinuum
2.	Strukturen und Prozesse der intersektoralen Delirprävention
3.	Informationsaustausch im Versorgungskontinuum
4.	Herausforderungen im Versorgungskontinuum
5.	Idealer Prozess der Versorgung im Kontinuum

### Maßnahmen der Früherkennung von Risikopatient:innen

In den Interviews zeigt sich, dass geriatrische Risikopatient:innen für operative Eingriffe bekannt sind, je nach Disziplin und „point of care“ (PoC) aber spezielle Faktoren zur Einschätzung des Delirrisikos hervorgehoben werden. So werden von der Allgemeinmedizin Menschen mit Demenz und ältere Erwachsene mit einer Alkohol- oder Benzodiazepinabhängigkeit angeführt. Vonseiten der Pflege und der Geriater:innen ist ein Alter über 75 Jahre als entscheidend anzusehen. Die Pflegeanamnese erfülle ebenso die Funktion, ein „vollständiges Bild“ zu erhalten, Geriater:innen beziehen auch die Biografie der Patient:innen ein. Aus Sicht der Anästhesist:innen relativiert das Fehlen einer aus dem Delirrisiko ableitbaren Maßnahme auf die Behandlung die Risikoeinschätzung des Delirs.

„Das Problem ist, […] wenn man sich den Versorgungsprozess als Pfeil vorstellt, dann bin ich [Anm.: Anästhesie] vielleicht Mitte-Ende. Da sind alle Entscheidungen schon getroffen. Das heißt also, in der Steuerung habe ich de facto keinen Einfluss, und ich bekomme die Patienten und muss mich dann einfach um sie kümmern.“ (AT_AN_I1, 14)

Für die Einschätzung eines Delirrisikos werden standardisierte Instrumente, aber auch ein Expertenblick benannt. In der Geriatrie ist das Screening eines Delirrisikos eingebettet in ein umfangreicheres geriatrisches Assessment, was das Delir in einen Komplex mit anderen Risikofaktoren des Alters wie Frailty, Demenz oder Immobilität bringt. Allgemeinmediziner:innen beschreiben kein gezieltes Screening nach Risikopatient:innen, auch aus Ressourcengründen, aber das Wissen über die Patient:innen sowie Aufzeichnungen über bereits stattgehabte Delire ermöglichen ein „Gefühl“ für die Einschätzung eines POD-Risikos.

### Strukturen und Prozesse der intersektoralen Delirprävention

Thematisiert werden Strukturen und Prozesse, die sich räumlich am Übergang vom intra- zum extramuralen Bereich sowie zeitlich von der prä- zur postoperativen Phase festmachen lassen. Anästhesist:innen beschreiben sich als „nicht verschränkt“ mit dem extramuralen Bereich und geben an, intramural nicht an der präoperativen Risikoeinschätzung eines Delirs mitzuwirken. Auch Geriater:innen sehen im Austausch mit dem extramuralen Bereich keine Strukturen oder regelhaften Prozesse zur Delirprävention. Prä- und postoperativ finden gelegentlich telefonische Anfragen und Auskünfte statt. Intramural werden Geriater:innen in der Regel präoperativ nicht in die Delirprävention involviert. Der Miteinbezug und die Verlegung auf die Geriatrie finden postoperativ bei jenen Patient:innen, die ein POD im Zuge des Krankenhausaufenthaltes entwickelt haben, statt.

„Was das Delir betrifft, da ist unser [Anm.: Geriatrie] erster Kontaktpunkt wenn die Patienten bei uns auf die Station kommen. Wir haben sehr wenig Kontakte zum extramuralen Bereich. Ein solcher ist nur gegeben, wenn jemand anruft aus dem niedergelassenen Bereich.“ (AT_GE_I9, 22)

Vonseiten der Allgemeinmediziner:innen wurde die fehlende regelhafte Einbindung in die Strukturen und Prozesse der intersektorale Delirprävention als zentrales Thema benannt. Als spezieller Beitrag der Allgemeinmediziner:innen in der Region Salzburg wird jedoch die präoperative Untersuchung (PROP) beschrieben. Wobei das PROP als ein allgemeines Assessment anzusehen ist, in dem implizit für das Delir relevante Risikofaktoren miterhoben werden, die nachfolgend im Krankenhaus in der elektronischen Patientenakte aufscheinen.

### Informationsaustausch im Versorgungskontinuum

In beiden untersuchten Regionen werden Informationslücken im Versorgungskontinuum erlebt, insbesondere an der Schnittstelle vom intra- zum extramuralen Bereich, vor als auch nach der Entlassung aus dem Krankenhaus. In den Interviews kommt zum Ausdruck, dass Informationen zu den Risikofaktoren im Versorgungskontinuum nicht systematisch weitergegeben werden. Geriater:innen kommunizieren mit dem extramuralen Bereich meist telefonisch. Sehr gewünscht wäre eine übergreifende und strukturierte Kommunikation, da die jeweiligen Disziplinen über je unterschiedliche Informationen, die an anderen PoC ebenso von Relevanz wären, verfügen.

„Mail-Verkehr, ja ginge, aber es ist auch nicht die optimale Lösung. Ich muss sagen, ich rede sehr gern mit den Kollegen direkt und persönlich, weil dann kann man auch sofort eine Lösung besprechen.“ (IT_AM_I4, 10)

Da die „Patient:innen viele Bedürfnisse mitbringen“, würden sich Geriater:innen auch eine „funktionierende Schnittstellenkommunikation“ mit den Angehörigen wünschen. Allgemeinmediziner:innen berichten, im Entlassungsschreiben über das im Krankenhaus aufgetretene POD zu lesen. Wobei im Entlassungsbrief über ein stattgefundenes Delir meist „nur als Nebendiagnose“ oder gar nicht berichtet wird. Allgemeinmediziner:innen aus der Region Südtirol berichten, dass sie „kein Feedback“ erhalten, ob das Arztschreiben im Krankenhaus gelesen wird.

Als veränderungswürdig beschreiben die HCP unisono das Fehlen eines für alle zugänglichen Dokuments, in dem ein Hinweis auf ein bereits in der Vergangenheit „durchgemachtes Delir“ gegeben wird.

„Es wäre gut, ein durchgemachtes Delir der Patienten irgendwo zu dokumentieren, um den nachfolgenden extramuralen oder stationären Bereich zu informieren, dass ein erhöhtes Risiko besteht.“ (AT_PF_I2_30)

### Herausforderungen im Versorgungskontinuum

Als Herausforderungen werden neben den fehlenden regionalen Strukturen, Schnittstellen und Verinselungen auch Unzulänglichkeiten an einzelnen PoC im Versorgungskontinuum von älteren delirgefährdeten Patient:innen angeführt. So kritisieren Anästhesist:innen die „nichtfunktionierende“ präoperative Abklärung im niedergelassenen Bereich. Demgegenüber merken Pflegende an, dass Anästhesist:innen und Chirurg:innen das Risiko eines POD anhand zu weniger Parameter einschätzen. Allgemeinmediziner:innen wiederum bemängeln, dass Chirurg:innen und Anästhesist:innen im Krankenhaus für die Weitergabe von Informationen oder Rückfragen zum POD nicht erreichbar wären.

„Die Kollegen im Krankenhaus zu kontaktieren, ist eine Herausforderung. Und manchmal lässt man es bleiben, weil man effektiv dann die Zeit nicht hat oder dann die Kollegen nicht erreichbar sind.“ (IT_AM_I4, 10)

Dass Informationen im Krankenhaus „neu gesammelt“ werden müssen, ist eine Konsequenz daraus, und ein Umstand, der die ohnehin knappen zeitlichen Ressourcen aller HCP zusätzlich belastet. Sehr gewünscht wäre also eine strukturierte Form der Zusammenarbeit zwischen intra- und extramuralen Bereich.

### Idealer Prozess der Versorgung im Kontinuum

Als wünschenswert wird in beiden Regionen ein Dokument beschrieben, in dem ein bereits in der Vergangenheit stattgefundenes Delir vermerkt ist, und die Risikofaktoren eines POD würden darin von den einzelnen Disziplinen speziell erhoben, eingetragen und für die nachfolgenden PoC im Versorgungskontinuum als Information verfügbar gemacht werden.

„Der geriatrische Patient, der Delirrisikopatient braucht einen gesonderten Weg durch den Aufenthalt. […] Idealerweise würde ich es so sehen, dass der Patient bei Eintritt in die Klinik einen Delirpass mithat, wo der Hausarzt ein Risiko erkannt hat, der [Patient] sozusagen ein Mascherl [Anm.: ein Label] bekommt für die Klinik, das besagt: ‚Patienten mit hohem Delirrisiko‘. Und der nachfolgende Pfad durch die Klinik weiß dann Bescheid.“ (AT_PF_I2_46)

Mithilfe eines Dokuments, das die Patient:innen im perioperativen Bereich begleitet, ein Risiko des POD ausweist und Versorgungspfade bestimmen würde, wären HCP auch besser auf ein POD vorbereitet und ein „zeitnahes Delirmanagement möglich“.

## Diskussion

Ziel dieser qualitativen Studie war es, die Erfahrungen in der intersektoralen und interprofessionellen Prävention des POD bei älteren Patient:innen aus Sicht der beteiligten HCP in den Regionen Salzburg und Südtirol zu beschreiben. Hauptergebnisse der Studie sind: die heterogene Identifikation von Risikopatient:innen, fehlende intersektoralen Strukturen und Prozesse der Kommunikation und Zusammenarbeit, sowie der Wunsch nach mehr interprofessioneller und interdisziplinärer Kommunikation.

### Identifikation von Risikopatient:innen

Beim Screening nach geriatrischen Risikopatient:innen werden je nach Disziplin und PoC unterschiedliche Faktoren zur Einschätzung des Delirrisikos als relevant hervorgehoben. Derzeit gibt es kein Vorhersagetool mit einheitlichen Risikofaktoren für ein Delirium, das als „Goldstandard“ empfohlen wäre [17]. Zu Validität und Reliabilität von Modellen, wie z. B. PROPDESC [8] oder PIPRA unter „Real-world“-Bedingungen gibt es noch kaum Daten [4]. Das Überschreiten einer Altersgrenze vonseiten der befragten Geriater:innen und Pflegenden wurde als wichtiger Risikofaktor genannt. Obwohl in Studien zur Prävention eines POD ein Alter ab 65 Jahren beschrieben wird [19], sind laut Einschätzung der interviewten Geriater:innen und Pflegenden primär die über 75-Jährigen als Risikopatient:innen in Betracht zu ziehen. Neben standardisierten Instrumenten aus der Literatur erfolgt die Einschätzung von Risikopatient:innen auch mittels Expertenblick. Ein Umstand, der auch in der Literatur beschrieben und mit persönlichen und strukturellen Faktoren in Verbindung gebracht wird [10]. Während die Verwendung valider und reliabler Assessmentinstrumente qua Leitlinie [3] empfohlen wird, zeigte eine aktuelle Untersuchung in 44 Ländern, dass 18,6 % der Befragten HCP angaben, für die Identifikation von Patient:innen mit Delir kein standardisiertes Instrument (z. B.: CAM) zu verwenden [11].

### Intersektorale Strukturen und Prozesse der Zusammenarbeit

Laut befragten HCP sind intersektorale Strukturen und Prozesse zur Delirprävention bei älteren Patient:innen kaum vorhanden. Besonders hervorstechend sind dabei die fehlende regelhafte Einbindung von Allgemeinmediziner:innen. Zumindest in Salzburg gibt es mit der PROP und deren Eingliederung in die elektronischen Patientenakte eine strukturierte allgemeinmedizinische extramurale Präventionsmaßnahme. Die Durchführung eines hausärztlich-geriatrischen Basisassessment ab einem Alter von 70 Jahre [13] kam in der vorliegenden Untersuchung nicht zur Sprache. In Südtirol sind die Allgemeinmediziner:innen kaum und nicht strukturiert eingebunden. Ein eher unstrukturiertes und beiläufiges geriatrisches Assessment in der Hausarztpraxis, durchgeführt im Rahmen üblicher Konsultationen, beschreibt auch eine aktuelle Studie aus Deutschland [13]. Der Wunsch nach einer strukturierten Kommunikation und Zusammenarbeit zwischen intra- und extramuralem Bereich kommt in den Ergebnissen dieser Studie deutlich zum Ausdruck. Aus der spärlichen Literatur zu diesem Thema geht jedoch hervor, dass auch in anderen Regionen die Prozesse zwischen intra- und extramuralen Bereich nicht ausreichend strukturiert sind [16]. Trotz geplanter Untersuchungen in diese Richtung sind noch keine Daten verfügbar [9, 11].

### Interprofessionelle und interdisziplinäre Kommunikation

Informationen zu den Risikofaktoren werden im Versorgungskontinuum nicht systematisch weitergegeben, sowohl die Einweisung in als auch die Entlassung aus dem Krankenhaus betreffend. Dies hat zur Konsequenz, dass nicht nur die Prävention, sondern auch das Management des Delirs darunter leidet. Dass die Diagnose POD nicht konsequent dem nachfolgenden extramuralen Bereich kommuniziert wird, ist dabei kritisch zu betrachten, aber entspricht dem Ergebnis einer Studie aus Deutschland [16]. Die Notwendigkeit einer kommunikativen Brücke zwischen intra- und extramuralem Bereich ergibt sich auch aus den Ergebnissen von Sturm et al. [16]. Obwohl der Einsatz von Mehrkomponentenmaßnahmen wirksam ist, lässt sich nur schwer feststellen, in wie vielen und welchen Kontexten sie eingesetzt werden. Der Umgang mit einem POD erfordert einen kooperativen, ganzheitlichen Ansatz, der mit der routinemäßigen Identifizierung von Deliriumrisikopatient:innen idealerweise bereits in der präoperativen Phase beginnen sollte [17]. Zur Verbesserung der interprofessionellen Prävention des POD bei älteren Patientinnen wird von den HCP ein Dokument beschrieben, etwa in Form einer Checklist, in dem von den einzelnen PoC die Information zu Risikofaktoren konsequent im Versorgungskontinuum weiterkommuniziert werden. Ebenso könnte in diesem Dokument ein bereits in der Vergangenheit durchgemachtes Delir vermerkt werden. So könnten das Delirrisiko im Verlauf umfassender eingeschätzt und der Versorgungspfad individuell angepasst werden.

## Limitationen

Kritisch ist anzumerken, dass mehr Interviews in Salzburg durchgeführt wurden sowie nur die Perspektive der HCP und nicht das Erleben der älteren Patient:innen und Angehörigen erhoben wurde. Das Sampling betreffend ist anzumerken, dass keine Therapeut:innen, die ebenso Teil des interdisziplinären und -professionellen Delir-Teams sind, eingeschlossen wurden. Auch eine noch konsequenter verfolgte komparative Analyse hinsichtlich Unterschieden zwischen den untersuchten Regionen bzw. den Berufsgruppen und Disziplinen kann kritisiert werden, wenngleich dies nicht Ziel der Studie war. Der Einschluss zweier Versorgungsregionen aus Italien und Österreich und eine erste Sättigung der Daten erhöhen jedoch die Glaubwürdigkeit und die Generalisierbarkeit der Daten. Die Gütekriterien qualitativer Forschung betreffend konnte eine regelgeleitete und systematische Forschung sichergestellt werden. Von einer kommunikativen Validierung der Ergebnisse wurde jedoch Abstand genommen.

## Schlussfolgerungen

Das POD bei älteren geriatrischen Patient:innen stellt eine interprofessionelle und intersektorale Herausforderung dar. Durch die systematische Identifikation von Risikopatient:innen, beginnend im extramuralen Bereich, und eine Verbesserung der intersektoralen Zusammenarbeit und Kommunikation der beteiligten HCP könnte die Prävention des POD bei elektiven Eingriffen weiter verbessert werden. Eine Checklist, die ein 360°-Profil von geriatrischen Patient:innen unter Berücksichtigung der wichtigsten Risikofaktoren für POD ermöglicht, wird als eine Hilfe für alle an den verschiedenen PoC beteiligten HCP angesehen. Die Entwicklung einer solchen Checklist wird aktuell im Rahmen der Studie zum „Geriatrischen Delir-Pass (GeDePa)“ wissenschaftlich begleitet und deren praktischer Nutzen und Machbarkeit untersucht.

## Fazit für die Praxis

- Die systematische Identifikation von Risikopatient:innen und die Nutzung von intersektoralen Strukturen und Prozessen der Kommunikation und Zusammenarbeit können die Prävention des POD bei elektiven Eingriffen weiter verbessern.
- Empfehlenswert wäre eine gemeinsame sektorenübergreifende Checklist zur Identifikation der wichtigsten Risikofaktoren für ein POD bei geriatrischen Patient:innen.

## Supplementary Information

Elektronisches Supplement 1: Interviewleitfaden (Auszug)

## Data Availability

Daten, die die Ergebnisse dieser wissenschaftlichen Publikation stützen, sind auf begründete Anfrage beim korrespondierenden Autor erhältlich.
