# Supplementary material for: Multiprofessional perspectives on intersectoral collaboration for the prevention of postoperative delirium in older patients—A qualitative study
Source: Z Gerontol Geriatr. 2025 May 5;59(1):26–31. [Article in German] doi: 10.1007/s00391-025-02442-4 (PMC12823623; doi:10.1007/s00391-025-02442-4)
Supplement: Supplementary file 1 — Elektronisches Supplement 1: Interviewleitfaden (Auszug) [file 391_2025_2442_MOESM1_ESM.docx]

**Elektronisches Supplement 1: Interviewleitfaden (Auszug)**

| **Themenblock 1: Häufigkeit und Relevanz von Delir** | |
| --- | --- |
| **Leitfragen** | **Themen für Sondierungsfragen** |
| - Wenn Sie an Ihre Erfahrungen als *[Profession]* denken: Wie relevant ist ein Delir bei älteren Patient*innen in Ihrem Arbeitsalltag? - Wie berücksichtigen Sie im *[Setting]* in Ihren täglichen Routinen bei älteren Patient*innen (mit elektiven Eingriffen) das Thema Delir? - Wo sehen Sie in Ihrem Bereich zum postoperativen Delir bei älteren Patient*innen mit elektiven Eingriffen die größten Herausforderungen? - **OPTIONAL:** - Wo sehen Sie Potential zur Optimierung in Ihrem Bereich? | *Häufigkeit, Relevanz im Versorgungsalltag*  *Typische Maßnahmen und Abläufe*  *Delir als Priorität in der aktuellen Versorgung von älteren Patient*innen mit elektiven Eingriffen*  *Grundlegende Aspekte mit Optimierungspotential in der Versorgung* |

| **Themenblock 2: Risikofrüherkennung und versorgungspraktische Prozesse** | |
| --- | --- |
| **Leitfragen** | **Themen für Sondierungsfragen** |
| - Wenn Sie jetzt ganz allgemein an das intersektoralen Versorgungskontinuum von älteren Patient*innen mit elektiven Eingriffen denken (z.B. eine 70-jährige Frau die eine Hüftprothese erhält): - Inwiefern finden in Ihrem *[Setting]* bei älteren Patient*innen Maßnahmen zur Delir-Risikoeinschätzung statt?   - Was wird zur Prävention eines postoperativen Delirs unternommen? Welche Strukturen und Prozesse zur Prävention eines postoperativen Delirs sind regional zu benennen?   - An welchen Points of Care (z.B. Hausarzt, Facharzt, Anästhesie, Pflege) werden aktuell Maßnahmen zur Früherkennung gesetzt?   - Wie gestaltet sich der Informationsaustausch zwischen den Sektoren bzw. den Settings oder Professionen, die eine Rolle spielen in dem Versorgungskontinuum?   - Welchen Gesundheitsprofessionen sind aus Ihrer Sicht im intersektoralen Versorgungsprozess zur Prävention des postoperativen Delirs zentral?   **VERTIEFUNG**   - Welche Herausforderungen erkennen Sie im intersektoralen Versorgungsprozess zur Prävention des postoperativen Delirs bei älteren Patient*innen mit elektiven Eingriffen? Und welche Potentiale zur Optimierung würden Sie vorschlagen? - Wie könnte ein idealer Prozess zur Prävention des postoperativen Delirs bei elektiven Eingriffen bei älteren Patient*innen aussehen? | *Konkret eingesetzte Maßnahmen: Identifikation, Assessments, Screenings, zentrale Risikofaktoren, Geriatrisches Assessment*  *Interprofessionelle Zusammenarbeit, Schnittstellen, Kommunikation, Informationsfluss*  *Informationen von und Support für Angehörige* |
| *[Setting]: Point-of-Care: Hausärztliche Praxis, klinikinterne geriatrische/chirurgische Abteilung*  *[Professionen]: Hausärztin/Hausarzt, Geriater/Geriaterin, Angehörige des gehobenen Dienstes für Gesundheits- und Krankenpflege/Diplomierte Pflegeperson, Anästhesist/Anästhesistin* | |
